# BCR::ABL1 levels at first month after TKI discontinuation predict subsequent maintenance of treatment‐free remission: A study from the “GRUPPO TRIVENETO LMC”

**DOI:** 10.1002/cam4.5158

**Published:** 2022-10-08

**Authors:** Sara Di Giusto, Eleonora Toffoletti, Massimiliano Bonifacio, Gianni Binotto, Maria Cristina Miggiano, Elisabetta Calistri, Manuela Stulle, Anna Ermacora, Rossella Stella, Luigi Scaffidi, Fabio D'Amore, Giorgia Scotton, Davide Griguolo, Giovanna De Matteis, Roberta Bertorelle, Mauro Krampera, Gianpietro Semenzato, Renato Fanin, Daniela Damiani, Mario Tiribelli

**Affiliations:** ^1^ Division of Hematology and BMT Azienda Sanitaria Universitaria Friuli Centrale Udine Italy; ^2^ Department of Medical Area University of Udine Udine Italy; ^3^ Department of Medicine, Section of Hematology University of Verona Verona Italy; ^4^ Department of Medicine, Hematology and Clinical Immunology University of Padua Paduva Italy; ^5^ Department of Hematology San Bortolo Hospital Vicenza Italy; ^6^ Hematology Unit Ca’ Foncello Hospital Treviso Italy; ^7^ Hematology Unit Azienda Sanitaria Universitaria Giuliano‐Isontina Trieste Italy; ^8^ Division of Internal Medicine Azienda Ospedaliera S. Maria Angeli Pordenone Italy; ^9^ Department of Life Sciences, Section of Biochemistry University of Verona Verona Italy; ^10^ Immunology and Molecular Oncology Istituto Oncologico Veneto Padua Italy

## Abstract

We analyzed BCR::ABL1 expression at stop and in the first month after discontinuation in 168 chronic myeloid leukemia patients who stopped imatinib or 2nd generation tyrosine kinase inhibitors (2G‐TKIs) while in sustained deep molecular response. Patients were divided among those who maintained response (group 1, *n* = 123) and those who lost major molecular response (group 2, *n* = 45). Mean BCR::ABL1 RNA levels 1 month after discontinuation were higher in group 2 than in group 1 (*p* = 0.0005) and the difference was more evident 2 months after stop (*p* < 0.0001). The same trend was found both for imatinib and 2G‐TKIs. A receiver operating characteristic (ROC) analysis to determine a threshold value of BCR::ABL1 at 1 month after discontinuation identified a cut‐off value of 0.0051%, with 92.2% specificity, 31.7% sensitivity and a likelihood ratio of 4.087.

## Graphical abstract

We analyzed BCR::ABL1 expression at stop and in the first month after discontinuation in 168 CML patients stopping imatinib or 2G‐TKIs, dividing patients who maintained response (1, *n* = 123) and those who lost MMR (group 2, *n* = 45). Mean BCR::ABL1 RNA levels at 1 and 2 months after discontinuation was higher in group 2 than in group 1, with a similar trend for imatinib and 2G‐TKIs. A BCR::ABL1 value <0.0051% at 1 month after discontinuation was the stronger predictor of treatment‐free remission (92.2% specificity).

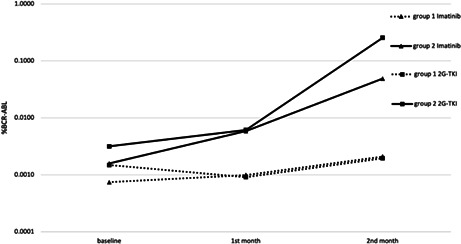

The chance to stop tyrosine kinase inhibitor (TKI) therapy while maintaining molecular response, the so called “treatment‐free remission” (TFR), has become common clinical practice and a goal in chronic myeloid leukemia (CML) patients.^1^ It is now agreed to attempt TFR in patients with a sustained deep molecular response (DMR), defined as a BCR::ABL1 transcript level ≤0.01% (MR^4^) or ≤0.0032% (MR^4.5^) in keeping with the International Scale, and resume TKI in case of loss of major molecular response (MMR).^2^, ^3^ The reported TFR rates, attained with diverse TKIs and according to slightly different definitions, range between 30% and 70%.^4^, ^5^, ^6^, ^7^ If factors that may predict a successful TFR are still poorly defined, though longer durations of TKI treatment and DMR have been associated with a higher chance of maintaining MMR,^6^ even fewer data are available on possible determinants to early detect patients who will eventually relapse after TKI stop.

This study was designed to evaluate BCR::ABL1 levels in patients attempting TFR in the clinical practice, to investigate if baseline BCR::ABL1 values and/or trends of transcript after TKI suspension could predict CML recurrence, conventionally defined as loss of MMR. BCR::ABL1 RNA expression was evaluated at TKI discontinuation (baseline), monthly during the first 6 months, bi‐monthly between 6 and 12 months and three‐monthly thereafter. DMR was defined as MR^4^ or deeper, undetectable (UND) transcript as a value of 0 BCR::ABL1 copies with a minimum of 10,000 ABL1 copies. The study was approved by the institutional review board (IRB) of the Department of Medical Area of the University of Udine (identifying number: 029/2020).

We retrospectively analyzed molecular data from 168 CML patients, followed at seven Hematology Units, that stopped imatinib (*n* = 112) and 2nd generation (2G) TKIs (*n* = 56) while in sustained DMR. All patients attempting TFR and for whom results of molecular analysis at baseline and in the first month after discontinuation were available were included in the study. Patients were divided in two groups; those who maintained MMR (group 1, *n* = 123) and those who lost MMR and were candidate to restart therapy for MMR loss (group 2, *n* = 45). Median time from TKI stop and loss of MMR and to restart of TKI therapy in group 2 were 4.0 months (range: 2.0–30.0) and 4.4 months (range: 2.8–31.7), respectively. For statistical analysis we used the Fisher exact test and the Mann–Whitney Wilcoxon test for non‐parametric distributions.

Main features of the two groups are summarized in Table 1. The two cohorts were comparable for CML features at diagnosis, TKI used to achieve stable DMR, duration of total TKI treatment and of stable DMR.

**TABLE 1:** Clinical and biological characteristics of CML patients who discontinued TKI treatment and that subsequently maintained (Group 1) or lost (Group 2) MMR

Characteristics	Group 1 (*n* = 123)	Group 2 (*n* = 45)	*p* Value
Age at CML diagnosis, years, median (range)	51.7 (18.5–80.6)	54.1 (17.1–79.2)	*0.53*
Male sex, no. (%)	67 (54.5)	25 (55.6)	*1.00*
Sokal risk score, no. (%)			
Low	68 (55.3)	22 (48.9)	*0.57* ^a^
Intermediate	43 (35.0)	17 (37.8)	
High	7 (5.7)	4 (8.9)	
Unknown	5 (4.0)	2 (4.4)	
BCR::ABL1 transcript type, no. (%)			
e13a2	29 (23.6)	12 (26.6)	*0.14* ^b^
e14a2	75 (61.0)	21 (46.7)	
e13a2 + e14a2	4 (3.2)	3 (6.7)	
other/unknown	15 (12.2)	9 (20)	
TKI therapy at stop, no. (%)			
imatinib	85 (69.1)	27 (60)	*0.36*
2G‐TKI (dasatinib, nilotinib)	38 (30.9)	18 (40)	
Duration of TKI therapy, month, median (range)	117.9 (28.9–209.4)	111.4 (33.7–181.9)	*0.97*
Duration of DMR, month, median (range)	82.8 (15.4–161.3)	68.6 (15.6–142.3)	*0.68*
BCR::ABL1 transcript at TKI stop, mean ± SD	0.0010 ± 0.0020	0.0018 ± 0.0029	*0.052*

^a^ Low versus other.

^b^ e14a2 versus other.

Mean BCR::ABL1 RNA expression at drug discontinuation for groups 1 and 2 was 0.0010 ± 0.0020 and 0.0018 ± 0.0029, respectively (*p* = 0.052); difference in molecular response at stop did not reach statistical significance neither in the imatinib cohort (group 1: 0.0007 ± 0.0019, group 2: 0.0016 ± 0.0026; *p* = 0.08) nor in the 2G‐TKI‐treated patients (group 1: 0.0015 ± 0.0023, group 2: 0.0022 ± 0.0032; *p* = 0.64). Also considering patients with UND BCR::ABL1 we found no differences between group 1 (85/123, 69%) and group 2 (25/45, 60%; *p* = 0.27).

Mean BCR::ABL1 RNA levels 1 month after discontinuation were significantly higher in group 2 (0.0060 ± 0.0107) than in group 1 (0.0010 ± 0.0026; *p* = 0.0005); this difference was confirmed and became even more evident 2 months after TKI stop as mean BCR::ABL1 value was 0.1354 ± 0.4259 in group 2 compared to 0.0020 ± 0.0076 in group 1 (*p* < 0.0001) (Figure 1A). Considering UND patients, we observed a highly significant prevalence in group 1 than in group 2 both at 1 month (83 vs. 16, *p* = 0.0004) and at 2 months (76 vs. 7, *p* < 0.0001).

**FIGURE 1:**
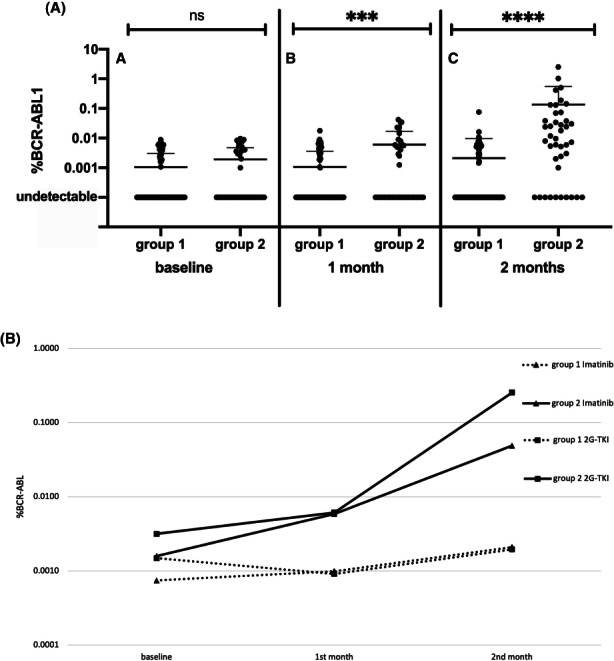
(A) Mean BCR::ABL1 RNA expression at drug discontinuation, one and 2 months after TKI stop in patients who maintained TFR (Groups 1) and in those who lost MMR (Group 2). (B) Slopes obtained with the mean values at baseline, one and 2 months after TKI discontinuation in patients who maintained TFR (Groups 1) and in those who lost MMR (Group 2) divided according to treatment (imatinib or 2G‐TKI).

The same trend was found in the imatinib group at month 1 (group 1: 0.0010 ± 0.0028, group 2: 0.0059 ± 0.0106; *p* = 0.003) and at month 2 (group 1: 0.0021 ± 0.0088, group 2: 0.0489 ± 0.0913; *p* < 0.0001) and in the 2G‐TKI group at month 1 (group 1: 0.0009 ± 0.0020, group 2: 0.0020 ± 0.0037; *p* = 0.014) and at month 2 (group 1: 0.0061 ± 0.0113, group 2: 0.2707 ± 0.6621; *p* = 0.0001). In the imatinib cohort, UND patients after 1 month were 60/85 in group 1 and 10/27 in group 2 (*p* = 0.004) and 54/85 and 4/27 at 2 months (*p* > 0.0001). Among patients stopping a 2G‐TKI, these figures were 23/38 versus 6/18 at 1 month (*p* = 0.11) and 22/38 and 3/18 at 2 months (*p* = 0.009).

Moreover, we graphed the modifications of mean values of BCR::ABL1 transcript at baseline, month 1 and month 2 in groups 1 and 2 divided for imatinib and 2G‐TKI (Figure 1B). The slopes of transcript modification were different between the 2 groups: mean change in BCR::ABL1 from baseline and month 2 was 0.0006 ± 0.0043 in group 1 versus 0.0237 ± 0.0450 in group 2; (*p* < 0.0001); a similar trend was evident both for imatinib (−0.0043 ± 0.0370 in group 1 vs. 0.0237 ± 0.0450 in group 2; *p* < 0.0001) and for 2G‐TKI (0.0002 ± 0.0018 in group 1 vs. 0.1132 ± 0.3057 in group 2; *p* = 0.0007).

To find out a threshold value of BCR::ABL1 RNA at 1 month after discontinuation, a ROC analysis was performed, defining an AUC = 0.6430 (CI 95%: 0.5361–0.7498; *p* = 0.0066): the cut‐off value for BCR::ABL1 was identified at 0.0051%. The chosen range has 92.2% specificity, 31.7% sensitivity, and a likelihood ratio of 4.087. In group 1, only 9 out of 113 evaluable patients (8.0%) had a BCR/ABL transcript >0.0051% at 1 month, compared to 13 out of 41 patients (31.7%) in group 2 (*p* = 0.005).

One of the faces of success of TKI therapy is the possibility, for a significant proportion of CML patients, to achieve a stable DMR and, therefore, try treatment discontinuation. However, identification of patients most likely to maintain TFR is of greatest interest, also considering the psychological impact of TKI stop^8^ and the efforts and costs required by a more frequent molecular monitoring. Different clinical trials, starting from the pioneering STIM1 study^4^ and subsequently multicenter and single institution experiences with imatinib or 2G‐TKIs^6^, ^9^, ^10^, ^11^, ^12^ found that duration of TKI therapy and of DMR were associated with an increased chance of successful TFR. In our experience, both length of treatment and of DMR were not different in the two groups. Similarly, we did not find any differences according to age, gender, Sokal risk score or BCR/ABL1 transcript type among the two cohorts. There was a non‐significant trend toward a lower BCR::ABL1 expression in patients subsequently maintaining TFR, though both cohorts had a mean level of MR4.5 or better, that a high number of patients with undetectable BCR::ABL1 in both groups could have had an impact on this finding and that, when evaluating separately, no differenced were found between the two groups in patients treated with imatinib or in those receiving 2G‐TKIs.

The significant raise in BCR::ABL1 transcript at the first month after discontinuation in patients subsequently loosing MMR confirms the finding from the Canadian group that showed that a shorter BCR::ABL1 doubling time was associated with higher rate of TFR failure.^13^ Our data suggest a similar trend in increasing BCR::ABL1 transcript for both imatinib and 2G‐TKIs. An absolute value of <0.0051% at 1 month was associated with an extremely high chance (over 90%) of maintaining MMR after treatment discontinuation.

It is arguable that more sensitive molecular techniques, such as digital polymerase chain reaction (PCR), could help identify at the time of TKI those stop patients who are more prone to relapse after discontinuation, particularly when BCR::ABL1 transcript is undetectable.^14^, ^15^ However, our data suggest that also the more widely available and standardized conventional PCR could rapidly identify patients with impending molecular recurrence.

Our data suggest that the chance of a successful TFR could be predicted already at 1 month after TKI discontinuation, both for patients stopping imatinib or 2G‐TKI. The 0.0051% BCR::ABL1 cut‐off value identify patients with the highest probability of maintaining MMR. This should not, at present, support a more “relaxed” molecular monitoring; while a BCR:ABL1 value <0.0051% at 1 month is strongly predictive of subsequent maintenance of TFR, still many patients with BCR::ABL1 value >0.0051% do not experience loss of MMR. If a lower transcript at 1 month could reassure, a higher value may suggest a more stringent monitoring in the first year (e.g., on a monthly basis).

## AUTHOR CONTRIBUTIONS

Mario Tiribelli designed the study. Massimiliano Bonifacio, Gianni Binotto, Maria Cristina Miggiano, Elisabetta Calistri, Manuela Stulle, Anna Ermacora, Rossella Stella, Luigi Scaffidi, Fabio D'Amore, Giorgia Scotton, Giovanna De Matteisand Roberta Bertorelle enrolled patients and contributed data. Sara Di Giusto and Eleonora Toffoletti performed experiments. Mauro Krampera, Gianpietro Semenzato, Renato Fanin, and Daniela Damiani supervised the project. Sara Di Giusto and Mario Tiribelli analyzed data and drafted the first version of the manuscript. All authors revised and approved the final version of the manuscript.

## CONFLICTS OF INTEREST

Mario Tiribelli reports personal fees (advisory board, speakers bureau) from Novartis, Bristol Myers Squibb, Pfizer and Incyte, all outside the submitted work. All other authors have nothing to disclose.

## Data Availability

For original data, please email the corresponding author.
